# The complete genome sequencing data of *Priestia aryabhattai* SPCL1 isolated from a heavy metal leachate-contaminated soil in Queretaro, México

**DOI:** 10.1016/j.dib.2026.112561

**Published:** 2026-02-07

**Authors:** Mario Eduardo Clemente Albores, María De Los Ángeles Hernández Tépach, Paola Itzel Herrera de la Torre, Mayra Paola Mena Navarro, María Carlota García Gutiérrez, Karla Isabel Lira De León, David Gustavo García Gutiérrez, Aldo Amaro Reyes, Miguel Angel Ramos López, José Alberto Rodríguez Morales, Erika Álvarez Hidalgo, Sergio de Jesús Romero Gómez, Juan Campos Guillén

**Affiliations:** a Facultad de Química, Universidad Autónoma de Querétaro, Cerro de las Campanas S/N, Querétaro 76010, México; b Center for Advanced Biomedical Research, School of Medicine, Autonomous University of Queretaro, Campus Aeropuerto Carretera a Chichimequillas S/N, Ejido Bolaños, 76140 Santiago de Querétaro, Qro, México; c Facultad de Ingeniería, Universidad Autónoma de Querétaro, Cerro de las Campanas S/N, Querétaro 76010, México

**Keywords:** *Priestia aryabhattai*, Genome sequencing, Antibiotic resistance genes (ARGs), Virulence factor genes, Heavy metal leachate-contaminated soil

## Abstract

We are providing the genome sequence of *Priestia aryabhattai* SPCL1, a bacterial strain isolated from a heavy metal leachate-contaminated soil in Querétaro, México. The Illumina NovaSeq platform was used to sequence the whole genome and the sequencing data obtained, including assembly and annotation, were analyzed on the BV-BRC platform. The genome, comprising 41 contigs and approximately 5.6 million base pairs with a GC content of 37.58 mol % and 6131 protein-coding sequences. In addition, 6 contigs of 146,177 bp (36.77 mol % *G* + *C*), 126,627 bp (33.27 mol % *G* + *C*), 16,881 bp (34.13 mol % *G* + *C*), 9835 bp (34.67 mol % *G* + *C*), 7402 bp (36.54 mol % *G* + *C*) and 4590 bp (35.38 mol % *G* + *C*) were assembled as plasmids. This analysis of genomic data represents a valuable resource for increasing knowledge of this bacterial specie and for possible applications in its biological functions. The genome data was deposited at National Center for Biotechnology Information (NCBI) under accession number Bioproject ID PRJNA1377581, Bio Sample ID SAMN53794006 and genome accession number ID JBSVDB000000000.

Specifications Table

**Table:** 

Subject	Biological sciences
Specific subject area	Microbiology, Genomics, Bioinformatics, Bioremediation
Data format	Raw, Filtered and analyzed
Type of data	Complete genome sequence in FASTA format Table(s) Figure(s)
Data collection	*Priestia aryabhattai* SPCL1 was isolated using Trypticase Soy Agar (TSA) medium from a heavy metal leachate-contaminated soil in Querétaro, México. Zymo Research DNA/RNA Shield reagent was used to preserve nucleic acids in the biological sample, then ZymoBIOMICS™DNA Miniprep Kit was used for DNA purification and sequenced with Illumina NovaSeq platform at Zymo Research, Irvine, CA, USA. For the bioinformatics analyses, the BV-BRC platform was used for adapter trimming, quality filtering, genome assembly and genome annotation. The Proksee platform was employed for the genome map, and the JSpeciesWS platform was used to assess species similarity via ANI and digital DNA-DNA hybridization (DDH) with Type (Strain) Genome Server (TYGS). The AMR phenotype analysis revealed resistance to: Ampicillin, Cephalothin, Cefotaxime, Dicloxacillin, Carbenicillin, Chloramphenicol, and Penicillin.
Data source location	Institution: Universidad Autónoma de Querétaro City/Town/Region: Querétaro, Qro. Country: México GPS coordinates: 20°35′28″N 100°24′36″O
Data accessibility	The assembly data is deposited in a public repository, and the analyzed data are presented in this report. Repository name: *Priestia aryabhattai* SPCL1 chromosome deposited in NCBI. Data identification number: JBSVDB000000000, Bio Project: PRJNA1377581, Bio Sample: SAMN53794006 Direct URL to data: https://www.ncbi.nlm.nih.gov/bioproject/PRJNA1377581 https://www.ncbi.nlm.nih.gov/biosample/SAMN53794006 https://www.ncbi.nlm.nih.gov/nuccore/JBSVDB000000000

## 1 Value of the Data

The complete genome sequence of *Priestia aryabhattai* SPCL1 may be important to increase the genetic background in this bacterial specie as well as understand the molecular adaptations to survive in soils contaminated with heavy metal leachates.

These data provide genomic information about heavy metal resistance mechanisms which could be employed in bioremediation.

Genome and plasmid data provides a valuable resource for comparative genome and plasmid studies with other similar bacterial species related.

Knowledge of genome versatility of this microorganism can be harnessed in various biotechnological applications, such as enzyme production, biopolymer synthesis, and bioremediation, even in agricultural applications such as biostimulants and biofertilizers.

## 2 Background

Leachate-contaminated soil has a variety of pollutants derived from municipal solid waste [1]. An important group of these contaminants are heavy metals, which have a high risk of affectation on the ecosystem [2]. Microbial communities have been studied in leachate-contaminated soils [[3], [4], [5]], but their physiological mechanisms to be employed in the bioremediation process in these leachate-contaminated soil remains to be explored. In this sense, the Gram-positive bacterium *Priestia aryabhattai* has been isolated from various ecological niches, mainly from soils and roots with important metabolic ability to be used [6]. Other members of this bacterial genus, for example have been evaluated in process of bioremediation in salinized soil [7], also as plant growth-promoting rhizobacteria [8], biosynthesis of small molecules and recombinant proteins [9] as well as ability to reduce As(V) and promote host plant growth [10]. In this respect, it is important to know whether bacteria isolated from heavy metal leachate-contaminated soil in Querétaro, México have potential functions to be characterized. Therefore, analyzing genome sequencing data of bacterial strains isolated from these contaminated soils can be essential to understand potential metabolic processes to be used in bioremediation systems. Currently, there is no available genomic information of microorganisms isolated from these local contaminated soils in Querétaro, México. In this study, we present the genome analysis of *P. aryabhattai* SPCL1 to provide valuable information associated with this heavy metal leachate-contaminated soil and contribute to the knowledge of this potential bacterial species to be employed in bioremediation.

## 3 Data Description

**Fig. 1:**
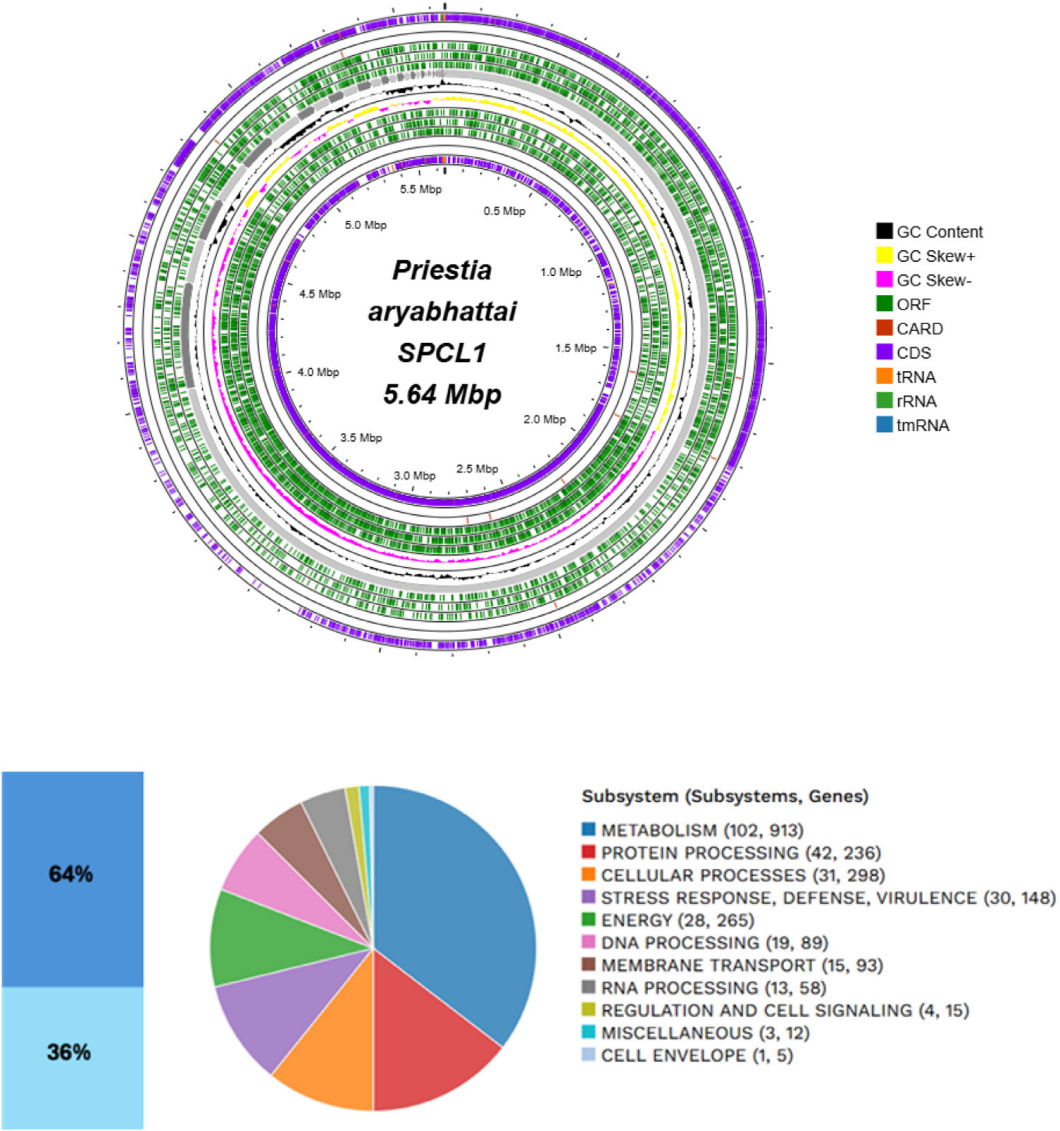
Circular genomic map and subsystems information for *P. aryabhattai* SPCL1. From the outside to the center are the assembled contigs, open reading frame (ORF), CDS in the front strand, CDS on the reverse strand, RNA genes, CDS with similarity to known antibiotic resistance genes, CDS with similarity to virulence factors, GC content and GC skew. In subsystems coverage, 64 % indicates a total of 3938 genes and 36 % represents those not indicated in subsystem average with a total 2193 genes.

Table 1 summarizes the genomic characteristics of *P. aryabhattai* SPCL1 obtained from the BV-BRC platform. The analysis resulted in a genome length of 5641,954 bp. The checkM integrity was 100 %, and contamination was 0.7 %. The genome sequence formed 41 contigs and an average *G* + *C* content of 37.58 mol %, with an N50 length (which is defined as the shortest sequence length at 50 % of the genome) of 4033,692 bp. The genome annotation shows 6131 protein-coding sequences (CDS), 4 ribosomal RNA (rRNA) genes, 97 transfer RNA (tRNA) genes, and other important features of the genome include 2193 hypothetical proteins, 10 virulence factors, 49 antibiotic resistance genes, 24 transporter genes, and 19 drug targets. Additionally, the distribution of subsystems in *P. aryabhattai* SPCL1, responsible for biological and metabolic processes important for the survival of the bacterium is described in Fig. 1. Six contigs were assembled as plasmids, and only five were related to plasmids detected in *P. megaterium* strains (Table 2, Table 3).

**Table 1:** Genomic description of *Priestia aryabhattai* SPCL1*.*

Characteristics	Source	Total
Genome Length	PATRIC	5641,954 bp
Number of contigs	PATRIC	41
Number of proteins characterized	PATRIC	6131
Number of putative/ hypothetical proteins	PATRIC	2193
Number of rRNA genes	PATRIC	4
Number of tRNA genes	PATRIC	97
Number of proteins with pathway annotation	KEGG	912
*G* + *C*	PATRIC	37.58 mol %
N50 contig size	PATRIC	4033,692 bp
Virulence factor	VFDB	1
Virulence factor	VICTORS	6
Virulence factor	PATRIC_VF	3
Antibiotic resistance genes	CARD	1
Antibiotic resistance genes	PATRIC	48
Transporter genes	TCDB	24
Drug target genes	DrugBank	19

**Table 2:** Contigs assembled as plasmids in *Priestia aryabhattai* SPCL1.

Genome features	Plasmid 1	Plasmid 2	Plasmid 3	Plasmid 4	Plasmid 5	Plasmid 6
Genome size	146,177 bp	126,627 bp	16,881 bp	9835 bp	7402 bp	4590 bp
GC contents	36.77 %	33.27 %	34.1 3 %	34.67 %	36.54 %	35.38 %
Total genes	179	176	25	14	12	9
CDSs	170	176	25	14	12	9
tRNA	9	0	0	0	0	0
Specialty genes	1	0	0	0	0	0

**Table 3:** Plasmids sequences that produce significant alignments with plasmids of *P. aryabhattai* SPCL1.

Plasmid	Sequence with significant alignment	Query cover	Percent identity	Length	Sequence ID
1	NO MATCH FOUND	NA	NA	NA	NA
2	*Priestia megaterium* strain PMUG01 plasmid pPMUG01–3	89 %	97.67 %	125,705 bp	CP150966.1
3	*Priestia megaterium* strain CK7 plasmid unnamed9	63 %	98.81 %	13,676 bp	CP085424.1
4	*Priestia megaterium* NCT-2 plasmid pNCT2_9	64 %	95.31 %	10,257 bp	CP032537.1
5	*Priestia megaterium* strain PMUG01 plasmid pPMUG01–5	38 %	93.62 %	6327 bp	CP150968.1
6	*Priestia megaterium* strain H2 plasmid unnamed15	42 %	88.69 %	10,024 bp	CP071481.1

**Fig. 2:**
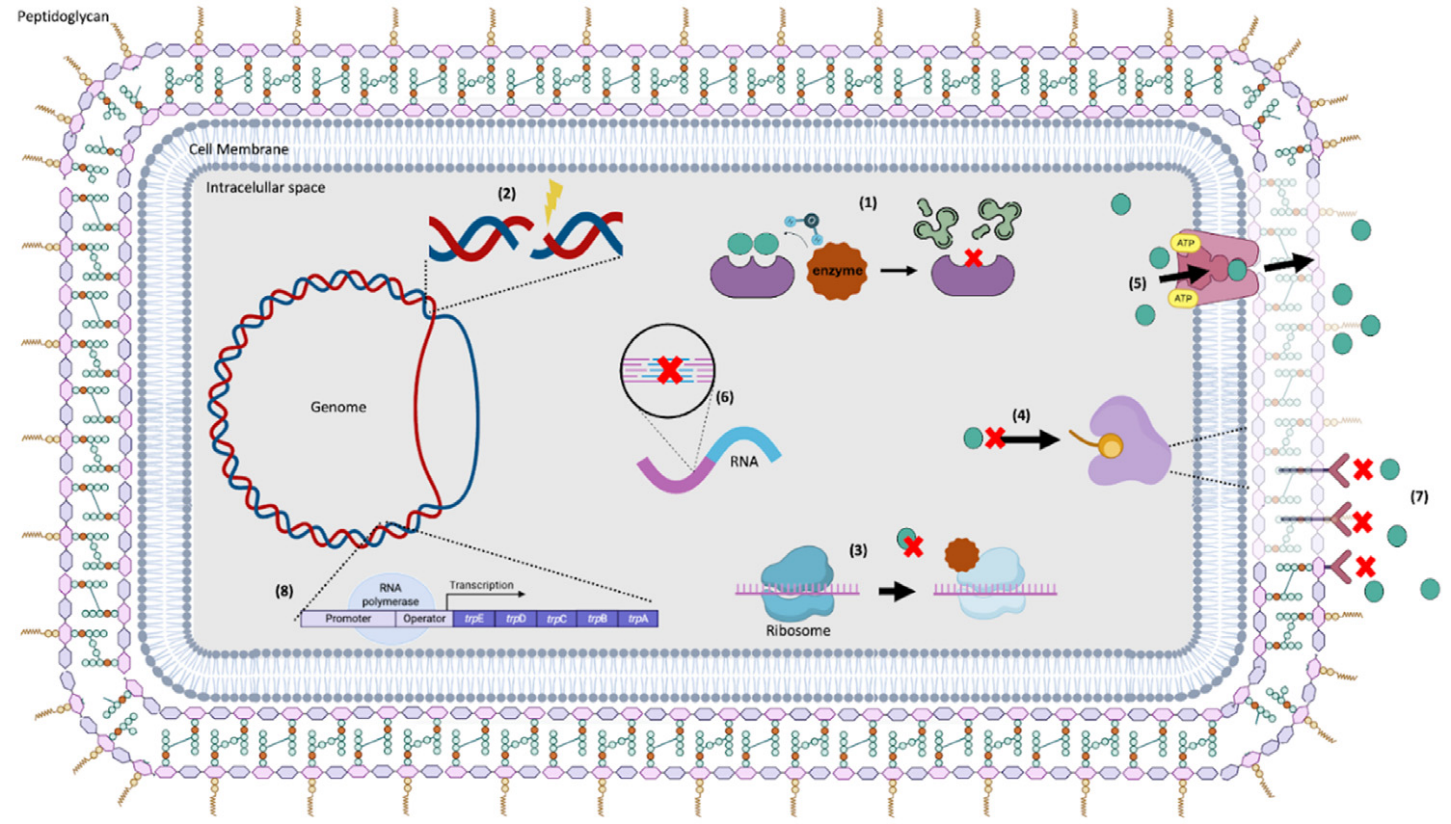
Mechanisms of antibiotic resistance predicted in 1) antibiotic inactivation enzyme 2) antibiotic target in susceptible species, 3) antibiotic target protection protein, 4) Antibiotic target replacement protein, 5) efflux pump conferring antibiotic resistance, 6) gene conferring resistance via absence, 7) protein altering cell wall charge conferring antibiotic resistance, 8) regulator modulating expression of antibiotic resistance genes. Created using Biorender under an academic licence*.*

Based on the genome analysis, eight mechanisms conferring resistance to the studied antibiotics were observed, as indicated in Fig 2. The mechanisms of action found in the isolates studied were: 1) antibiotic inactivation enzyme, associated with the presence of the *CatA* and *FosB* genes, 2) antibiotic target in susceptible species, associated with the presence of the *Alr, Ddl, dxr, EF-G, EF-Tu, folA, Dfr, folP, gyrA, gyrB, inhA, fabl, Iso-tRNA, kasA, MurA, rho, rpoB, rpoC, S10p* and *S12p* genes, 3) antibiotic target protection protein, associated with the presence of the *BcrC* gene, 4) Antibiotic target replacement protein, associated with the presence of the *fabL* gene, 5) efflux pump conferring antibiotic resistance, associated with the presence of the *BceA* and *BceB* genes, 6) gene conferring resistance via absence, associated with the presence of the *gidB* gene, 7) protein altering cell wall charge conferring antibiotic resistance, associated with the presence of the *GdpD* and *PgsA* genes, 8) regulator modulating expression of antibiotic resistance genes, associated with the presence of the *BceR, BceS, LiaF, LiaR* and *LiaS* genes.

Based on the bacterial virulence factors (VDFB) source, the virulence-related genes are shown in Table 4.

**Table 4:** Predicted virulence-related genes in *Priestia aryabhattai* SPCL1 identified through VFDB analysis.

Gen	Product	Mechanism of action
*recA*	*RecA* protein	DNA repair and homologous recombination in bacteria
*sodA1*	Superoxide dismutase [mn] (EC.1.15.1.1)	Catalyzes the disproportionation of superoxide into oxygen and hydrogen peroxide
*clpP*	ATP-dependent *ClpP* protease proteolytic subunit *ClpP*	Involved in the degradation of misfolded or damaged protein as well as in the degradation of regulatory proteins, affecting virulence, antibiotic tolerance and metabolism.
*codY*	GTP-sensing transcriptional pleiotropic repressor *CodY*	Represses gene expression or in some cases can activate gene expression
*clpX*	ATP- dependent Clp protease ATP- binding subunit *ClpX*	Protein coding
*EF1623*	Ethanolamine utilization protein similar to *PduA/Pduj*	A surface-anchored protein that promotes bacterial adhesion to host tissues by binding to host glycoconjugates like sialic acid
*purA*	Adenylosuccionate synthetase (Ec 6.3.4.4)	It is essential for the synthesis of purines in bacteria for their growth and survival. Alterations in these genes may be involved in antibiotic resistance mechanisms
*fur*	Ferric uptake regulation protein FUR	Its main function is to control the expression of genes involved in iron homeostasis and other processes, including virulences in pathogenic bacteria.
*purB*	Adenylosuccinate lyase (EC 4.3.3.3) @ SACAR lyase	Protein coding

**Fig. 3:**
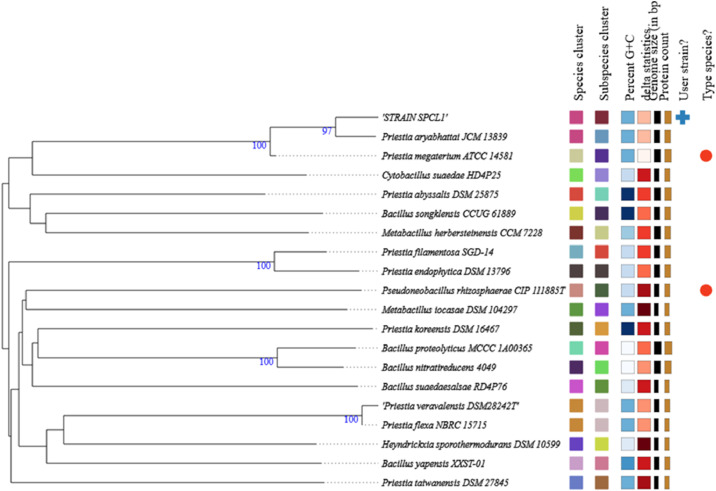
Phylogenomic tree based on TYGS result using SPCL1 genome data set. Tree inferred with FastME 2.1.456 from GBDP distances calculated from genome sequences. Branch lengths are scaled in terms of GBDP distance formula d5; numbers above branches are GBDP pseudo-bootstrap support values from 100 replications.

**Fig. 4:**
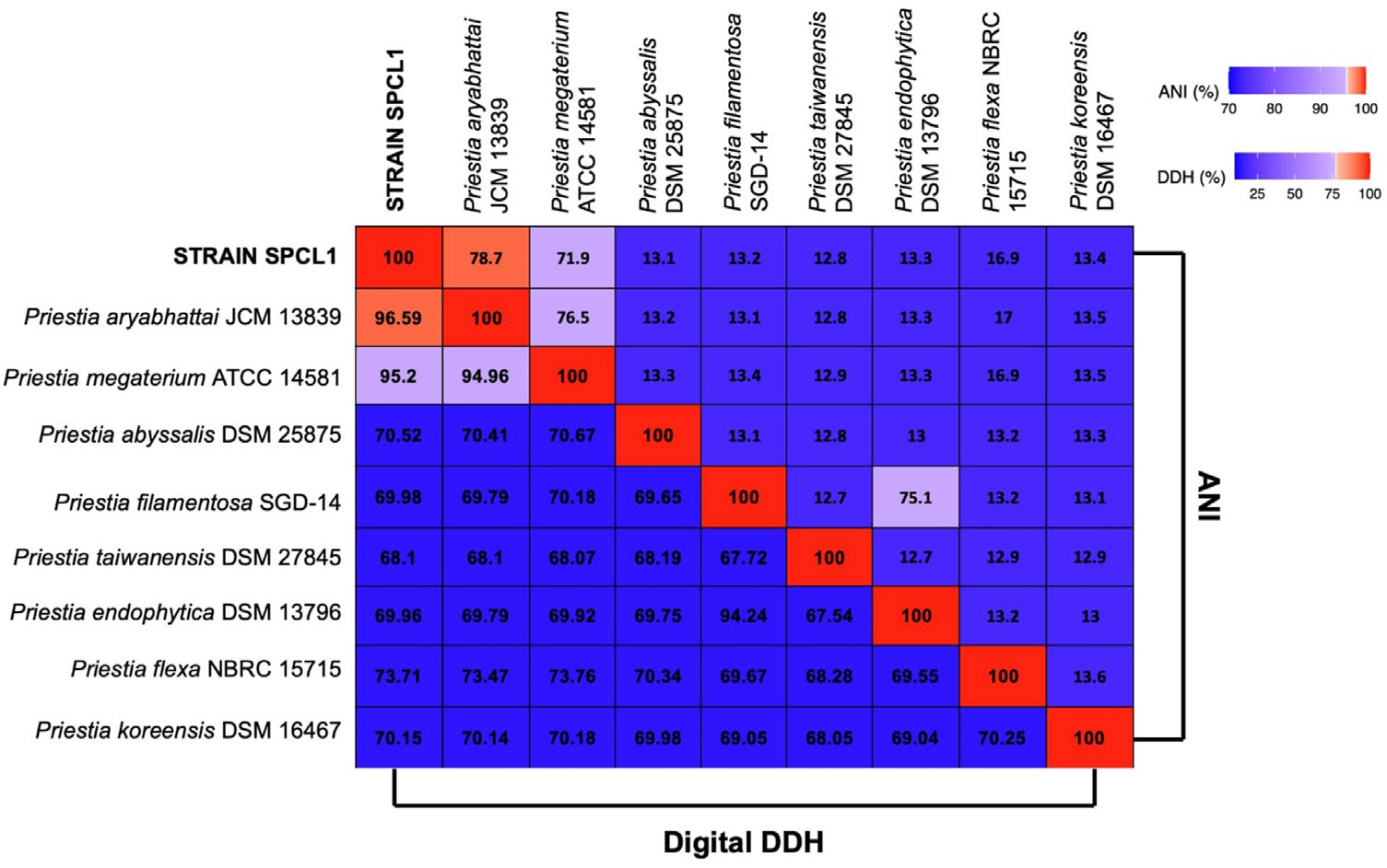
Heat-maps showing pair-wise average nucleotide identity (ANI) and digital DNA-DNA hybridization (DDH) values of strain SPCL1 and the closely related *Priestia* type strains.

The phylogenomic analyses is show in Fig. 3. The comparative pairwise analysis of the strain SPCL1 genome and type strains revealed ANI values of 96.59 % with *P. arybhattai JCM13839*, while DNA digital hybridization values revealed 78.7 % for the same strain (Fig. 4), which were higher that the thresholds (ANI, ~95 %; digital DDH, 70 %), confirming this classification as *P. arybhattai SPCL1.*

The analysis of the AMR phenotype performed on Mueller Hilton agar revealed resistance to ampicillin, cephalothin, cefotaxime, dicloxacillin, carbenicillin, chloramphenicol and penicillin. The bacterial strain showed sensitivity to the following antibiotics: tetracycline, sulfamethoxazole/trimethoprim, vancomycin, ciprofloxacin, clindamycin, amikacin, erythromycin, gentamicin, netilmicin, nitrofurantoin and norfloxacin. (Table 5).

**Table 5:** Resistance and sensitivity of the SPCL1 strain to different antibiotics.

Antibiotic	Phenotype	Antibiotic	Phenotype	Antibiotic	Phenotype
Ampicillin (10 µg)	R	Ciprofloxacin (5 µg)	S	Erythromycin (15 µg)	S
Cephalothin (30 µg)	R	Clindamycin (30 µg)	S	Gentamicin (10 µg)	S
Cefotaxime (30 µg)	R	Dicloxacillin (1 µg)	R	Penicillin (6 µg)	R
Tetracycline (30 µg)	S	Amikacin (30 µg)	S	Netilmicin (30 µg)	S
Sulfamethoxazole/ Trimethoprim (25 µg)	S	Carbenicillin (100 µg)	R	Nitrofurantoin (300 µg)	S
Vancomycin (30 µg)	S	Chloramphenicol (30 µg)	R	Norfloxacin (10 µg)	S

*S*=Sensitive; *R*=Resistant.

## 4 Experimental Design, Materials and Methods

### 4.1 Sample collection and microbial isolation

Soil sample was collected at depths of 10–30 cm from heavy metal leachate-contaminated soil in Queretaro, Mexico. The soil sample (1 g) was diluted in 10 mL of sterile water and vortexed by 5 min. Serial dilutions (10^−1^ to 10^−6^) were spreaded on Trypticase soy agar (TSA) medium (Difco Laboratories, Detroit, MI, USA) incubated at 37 °C for 24 h. The bacterial strain determined as SPCL1 was selected based on antibiotic resistance tested on TSA medium amended with Chloramphenicol (10 µg). Atomic absorption spectrometry (PerkinElmer AAnalyst 300) was used for heavy metal analysis in soil sample for arsenic (93.7 mg/kg), mercury (1284.7 mg/kg), cadmium (0.08 mg/kg), copper (4.9 mg/kg), chrome (12 mg/kg) and lead (6.38 mg/kg).

### 4.2 Genome sequencing, assembly and annotation

Genomic DNA from *Priestia aryabhattai* SPCL1 was extracted using the ZymoBIOMICS™ DNA Miniprep Kit and sequenced at Zymo Research with Illumina® Novaseq technology. The quality of fastq reads was performed at BV-BRC using the pipelines [11]; Trim Galore version 0.6.5dev with following parameters: Maximum trimming error rate: 0.1 (default), minimum required adapter overlap (stringency): 1 bp and minimum required sequence length for both reads before a sequence pair gets removed: 20 bp. Paired end reads were filtered with Fastq-Pair. The reads were *de novo*-assembled into contigs with SPAdes genome assembler v4.0.0. Then PATRIC, and RASTtk was used for genome annotation [11,12]. A circular genome map was generated using Proksee [13]; while species similarity was assessed with JSpeciesWS through average nucleotide identity (ANI) [14,15]; in addition, the digital DNA-DNA hybridization (DDH) analysis and Phylogenomic tree was constructed using the Type (Strain) Genome Server (TYGS) [16,17]. The plasmidSPAdes algorithm was used for assembling plasmids from assembled reads [18], then PlasmidScope platform was used for annotation [19], and finally plasmid database was used to find similar plasmids [20]. All software was run with default parameters.

### 4.3 Antibiotic sensitivity test

Antibiotic resistance and susceptibility in *Priestia aryabhattai* SPCL1 were evaluated by disk diffusion. Multidiscs for Grampositive (+) bacteria; ampicillin (10 μg), cefolatin (30 μg), cefotaxime (30 μg), ciprofloxacin (5 μg), clindamycin (30 μg), dicloxacycline (1 μg), erythromycin (15 μg), gentamicin (10 μg), penicillin (10 U), tetracycline (30 μg), sulfametazole / trimethoprim (25 μg), vancomycin (30 μg). Multidiscs for Gram negative (-) bacteria: amikacin (30 μg), ampicillin (10 μg), carbenicillin (100 μg), cephalothin (30 μg), cefotaxime (30 μg), ciprofloxacin (5 μg), chloramphenic (30 μg), gentamicin (10 μg), netilmycin (30 μg), nitrofurantoin (300 μg), norfloxacin (10 μg) sulfamethoxazole/trimetropim (25 μg).

## Limitations

Not applicable

## Ethics Statement

This work does not involve human subjects or animal subjects. The authors declare that this manuscript is original work and has not been published elsewhere.

## CRediT Author Statement

Mario Eduardo Clemente Albores: Writing-original draft, Methodology; María De Los Ángeles Hernández Tépach: Writing-original draft, Methodology; Paola Itzel Herrera de la Torre: Writing-original draft, Methodology; Mayra Paola Mena Navarro: Writing-original draft, Methodology; Maria Carlota García Gutiérrez: Writing-original draft, Methodology; Karla Isabel Lira De León: Writing- original draft, Resources, Methodology; David Gustavo García Gutiérrez: Writing-review & editing; Aldo Amaro Reyes: Writing-review & editing; Miguel Angel Ramos López: Writing-review & editing; José Alberto Rodríguez Morales: Writing-review & editing; Erika Álvarez Hidalgo; Conceptualization, Methodology; Sergio de Jesús Romero Gómez: Resources, Writing-review & editing; Juan Campos Guillen: Validation, Supervision, Resources, Writing-review & editing, Supervision.

## Data Availability

NCBIPriestia aryabhattai strain:spcl1 Genome sequencing (Original data)
